# Biphenylene-containing polycyclic conjugated compounds

**DOI:** 10.3762/bjoc.19.141

**Published:** 2023-12-13

**Authors:** Cagatay Dengiz

**Affiliations:** 1 Department of Chemistry, Middle East Technical University, 06800 Ankara, Turkey

**Keywords:** acenes, biphenylene, [N]phenylenes, polycyclic aromatic compounds

## Abstract

There has been a growing emphasis on the synthesis of polycyclic conjugated compounds, driven by their distinct structural characteristics that make them valuable candidates for use in cutting-edge technologies. In particular, acenes, a subgroup of polycyclic aromatic compounds, are sought-after synthetic targets due to their remarkable optoelectronic properties which stem from their π-conjugation and planar structure. Despite all these promising characteristics, acenes exhibit significant stability problems when their conjugation enhances. Various approaches have been developed to address this stability concern. Among these strategies, one involves the incorporation of the biphenylene unit into acene frameworks, limiting the electron delocalization through the antiaromatic four-membered ring. This review gives a brief overview of the methods used in the synthesis of biphenylenes and summarizes the recent studies on biphenylene-containing polycyclic conjugated compounds, elucidating their synthesis, and distinct optoelectronic properties.

## Introduction

**Figure 1:**
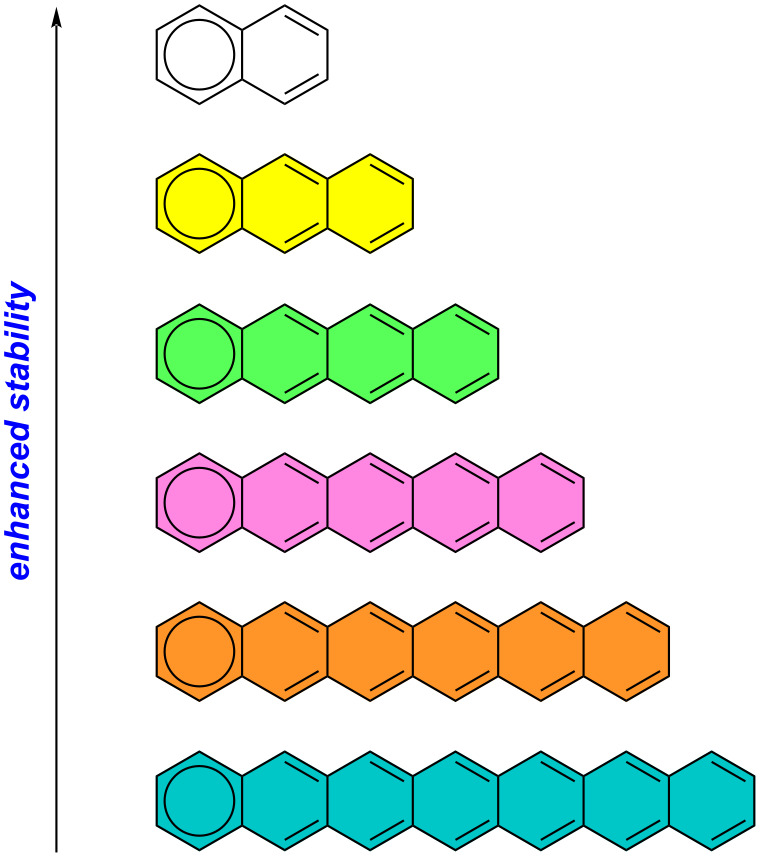
The correlation between stability and Clar's rule in acenes.

Acenes represent an important category of carbon-rich polycyclic aromatic hydrocarbons (PAHs) characterized by the presence of linearly fused benzene rings [1–2]. Investigating the electronic properties of acenes is essential for understanding the correlations between structure and electronic properties, as these units serve as fundamental building blocks in graphite and carbon nanotubes [3]. The limited stability of this particular class of PAHs arises as a key challenge, primarily attributed to their extended conjugation. The longer acenes exhibit increased reactivity, readily undergoing processes of oxidation and dimerization, consequently disrupting the molecular conjugation [4]. This instability poses a significant obstacle in their widespread application across various devices [4]. The decline in stability seen in larger acenes can be attributed to Clar's rule, which considers the increasing number of non-sextet rings throughout the acene series as a contributing factor (Figure 1) [5–6].

Numerous approaches have been developed to address the challenges arising from the instability and solubility issues encountered in acenes. These include the incorporation of heteroatoms within the acene backbone [7–8], stabilization of the acene core structure through the integration of diverse units [9–10], and the introduction of bulky substituents [11]. These approaches aim to maintain the desirable electronic properties of acenes while mitigating the aforementioned challenges to the best possible extent. Our focus in this review is primarily on exploring the role of biphenylenes in stabilizing the core structures of acenes and other PAHs.

## Review

### Biphenylenes and [N]phenylenes

**Scheme 1:**
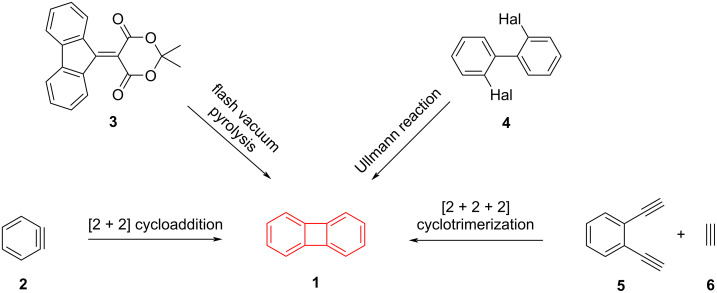
General synthetic strategies to access the biphenylene core **1**.

Biphenylene (**1**), which consists of two aromatic benzene rings connected with a four-membered ring, is a highly intriguing compound in terms of its structure. It possesses a planar configuration and consists of 4*n* π-electrons, rendering it antiaromatic. However, despite being antiaromatic, biphenylene is more stable than other known antiaromatic compounds [12]. Research using computational methods to investigate how benzo and benzocyclobutadiene annulations impact the ring current density of biphenylene derivatives reveals that the antiaromatic (paratropic) current density in the 4-ring structure can range widely, shifting from highly antiaromatic to nonaromatic limits based on the annulation modes employed [13–14]. Since Lothrop's initial successful synthesis of biphenylene in 1941 [15], numerous studies have been carried out, highlighting four prominent synthetic approaches. These methods include flash vacuum pyrolysis [16–18], [2 + 2] cycloaddition [19–20], [2 + 2 + 2] cycloaddition [21], and the Ullmann reaction [15,22] (Scheme 1). Due to the observed low yields in flash vacuum pyrolysis, the difficulty in synthesizing starting materials, such as **3**, and the impractical nature of scaling up the method for large quantities, the other three approaches have gained popularity for synthesizing biphenylene derivatives [23]. The utilization of in-situ aryne synthesis to generate biphenylene through the dimerization of arynes **2** from diverse substrates has gained popularity. However, this approach occasionally gives rise to the production of high-energy intermediates, such as benzenediazonium-2-carboxylate, and yields that are comparatively low [20]. After the Ullmann reaction was successfully employed for the first reported synthesis of biphenylene [15], subsequent studies have explored various transition-metal-mediated coupling reactions using 2,2'-dihalogenated biphenyls **4** as starting materials [24–25]. Although the cobalt-mediated alkyne trimerization route frequently used by Vollhardt and co-workers is not the first choice for the synthesis of the biphenylene itself, it has led to the synthesis of structurally demanding substituted biphenylenes and the emergence of a family of polycyclic hydrocarbons called [N]phenylenes.

**Figure 2:**
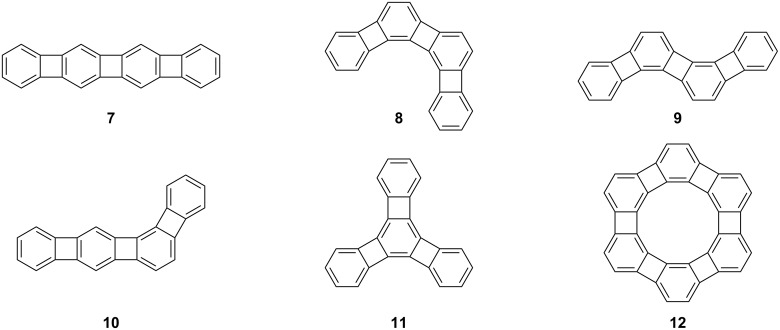
[N]Phenylenes **7**–**12** with different topologies.

The utilization of cobalt-mediated alkyne trimerization facilitated the synthesis of [N]phenylenes exhibiting diverse structural configurations, including linear **7**, angular **8**, zig-zag **9**, bent **10**, branched **11**, and cyclic **12** topologies (Figure 2) [26–30]. In [N]phenylene structures, the presence of a formally antiaromatic four-membered ring leads to the localization of π-electrons on the benzene rings [31]. Despite this phenomenon, the oligomer series demonstrates a decreasing band gap, indicating the ability of electrons to transmit through the four-membered rings [32].

### Phenylene-containing oligoacenes (POAs)

**Scheme 2:**
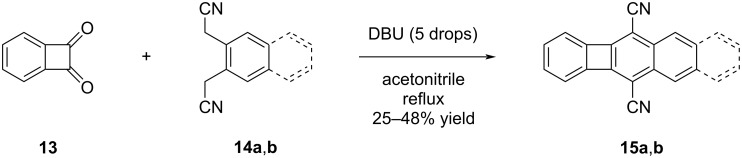
Synthesis of POAs **15a** and **15b** via reactions of BBD **13** and bis(cyanomethyl) compounds **14a** and **14b**.

The localization of π-electrons and the consequent decrease in band gaps observed in the [N]phenylene series have sparked interest in exploring acene–biphenylene hybrid structures. If this trend could be maintained in practical applications, it would offer the opportunity to retain the desirable electronic properties while mitigating the inherent stability concerns associated with acenes. The underlying principle guiding the design is to maximize stability by incorporating the largest possible number of Clar sextets, while concurrently minimizing any adverse impact on electronic properties resulting from reduced electron delocalization. In 1983, McOmie and co-workers reported the first synthesis of phenylene-containing oligoacenes (POAs) [33]. The primary objective of their work was to establish an alternative synthetic approach to the existing methods for biphenylene synthesis described in the literature, rather than focusing on the optoelectronic properties of the resulting compounds. Small quantities of the key starting material benzocyclobutene-1,2-dione (BBD, **13**) were obtained through the pyrolysis of indane-1,2,3-trione. When BBD **13** was subjected to reflux conditions with bis(cyanomethyl) compounds **14a** (a benzene derivative) and **14b** (a naphthalene derivative) in acetonitrile, the desired POAs **15a** and **15b** were obtained with yields of 25% and 48%, respectively (Scheme 2). It was also reported in the same study that the yield of **15b** increased up to 62% when the water formed during the reaction was removed with CaH_2_.

**Scheme 3:**
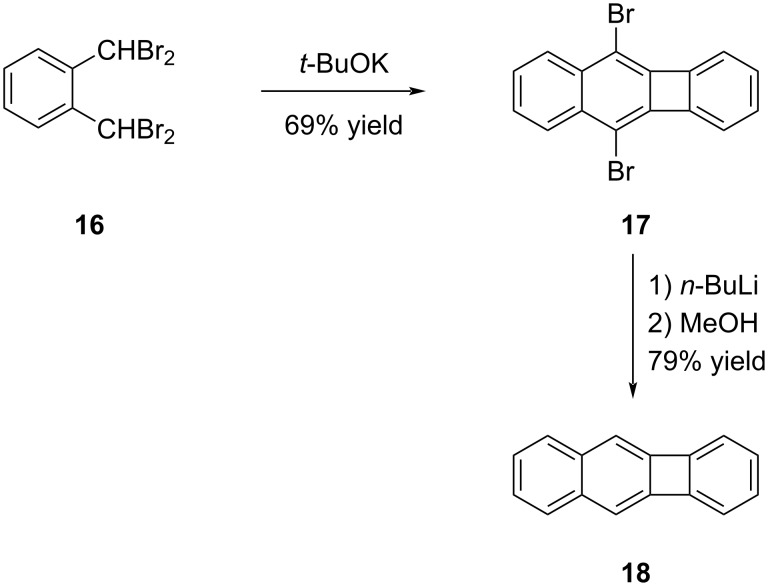
Synthesis of benzo[*b*]biphenylene (**18**).

The initial results on biphenylene and its more extensively fused counterparts led to another notable study conducted by Jensen and Coleman in 1959 [34]. By subjecting α,α,α′,α′-tetrabromo-*o*-xylene (**16**) to *t*-BuOK to anhydrous conditions, biphenylene derivative **17** was obtained with a yield of 69% (Scheme 3). In the final step of the synthesis, a halogen–lithium exchange was carried out, followed by treatment with MeOH, resulting in a 79% yield of benzo[*b*]biphenylene (**18**).

**Scheme 4:**
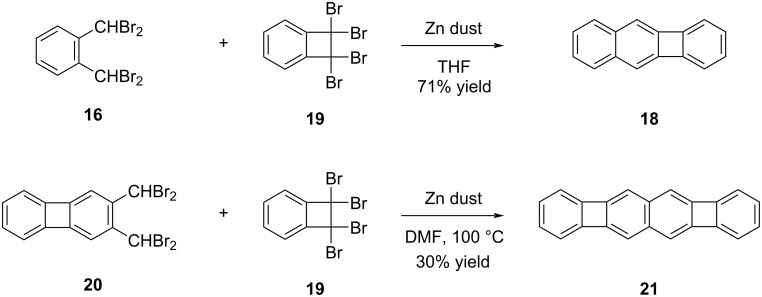
Synthesis of benzobiphenylene **18** and POA **21**.

In their efforts to find a more general method for the synthesis of benzo[*b*]biphenylenes, Barton and co-workers were able to synthesize benzo[*b*]biphenylene (**18**) in 71% yield by the reaction of equal molar amounts of tetrabromo compound **16** and 1,1,2,2-tetrabromo-1,2-dihydrobenzocyclobutene (**19**) in the presence of activated zinc dust in THF (Scheme 4) [35]. By making minor adjustments to the reaction conditions, such as employing DMF as the solvent and raising the temperature to 100 °C, along with utilizing compound **20** as the starting material, Barton and his team achieved the synthesis of POA **21**. This POA featured an extended conjugation and was obtained in 30% yield.

**Scheme 5:**
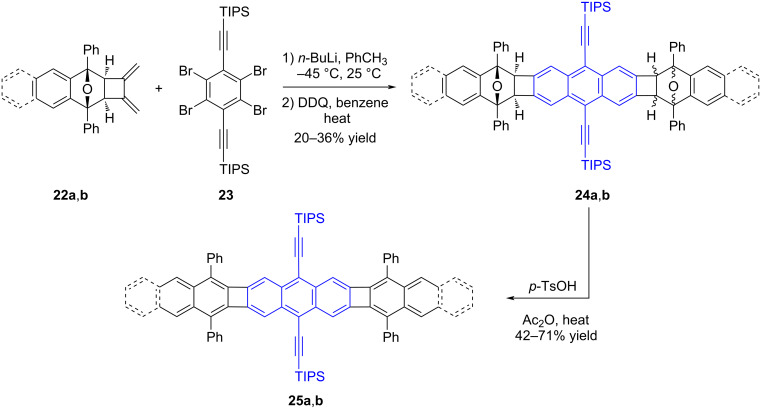
Synthesis of symmetric POAs **25a** and **25b**.

In a study released by Swager and Parkhurst in 2012 [36], the term POA has been used for the first time in the literature. In this study, they successfully synthesized POAs **25a** and **25b** by employing sequential Diels–Alder reactions (Scheme 5). Furthermore, the researchers conducted a comprehensive investigation into the optical and electrochemical characteristics of these compounds. The key component employed in the production process, known as 3,4-bis(methylene)cyclobutene, was generated through the application of flash vacuum pyrolysis to 1,5-hexadiyne. When 3,4-bis(methylene)cyclobutene undergoes a reaction with dienes like 1,3-diphenylisobenzofuran and 1,3-diphenylisonaphthofuran, it selectively produces compounds **22a** and **22b**. In the subsequent step, the exocyclic methylidene groups react with bisarynes, which are in situ formed from **23**, resulting in the formation of symmetric polycyclic structures **24a** and **24b**. These isomers obtained as a mixture are then subjected to treatment with *p*-TsOH in acetic acid, without the need for further purification, to yield the desired products **25a** and **25b** in 71 and 42% yields, respectively. When comparing compounds **25a** and **25b**, UV–vis and fluorescence studies (λ_max_ = 500 nm, λ_em_ = 502 nm, Φ_em_ = 0.45 for **25a**; λ_max_ = 513 nm, λ_em_ = 517 nm, Φ_em_ = 0.26 for **25b**; λ_max_ = 442 nm, λ_em_ = 444 nm, Φ_em_ = 0.97 for 9,10-bis((triisopropylsilyl)ethynyl)anthracene – blue-colored) provide clear evidence of a bathochromic shift and a reduction in the optical band gap. These results support the idea that the introduction of biphenylene linkages may decrease delocalization in the structure. However, despite this reduction, there is still communication between the acene units evident from the decreased band gaps in compounds **25a** and **25b**.

**Scheme 6:**
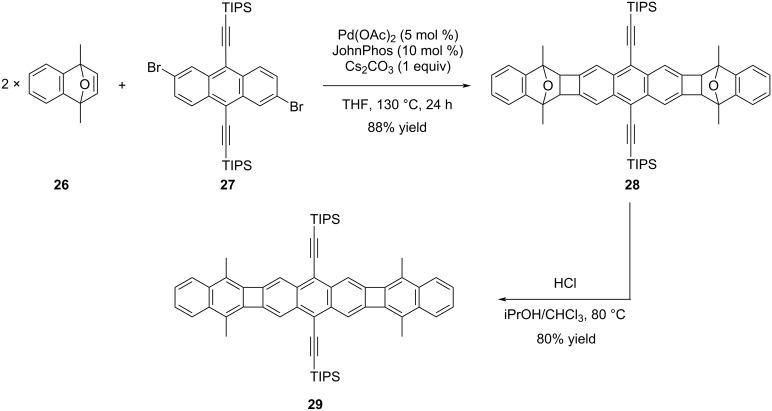
Synthesis of POA **29** via palladium-catalyzed annulation/aromatization reaction.

Despite the achievements in the synthesis of [N]phenylene and POAs, the challenges of low yield and stability issues, particularly with the starting materials employed in the syntheses, prompted researchers to explore alternative and improved methods. In this context, Xia and co-workers drew inspiration from their prior investigations on palladium-catalyzed annulation reactions concerning ladder polymers [37]. They envisioned that by making minor modifications to the starting materials, they could readily access structurally complex POAs. The researchers efficiently conducted palladium-catalyzed C–H activated annulation reactions, involving oxanorbornadiene derivative **26** and aryl bromides including dibromoanthracene **27** [38]. Subsequent aromatization reactions were then carried out, resulting in the successful synthesis of the target POAs with high yields. The study involved the synthesis of numerous polycyclic hydrocarbons containing both electron-withdrawing and -donating side groups. Among the various compounds synthesized, a particularly noteworthy achievement was the successful synthesis of acene-type compound **29** using 2,6-dibromo-9,10-bis(triisopropylsilylethynyl)anthracene (**27**), which was accomplished with high yields (Scheme 6).

The presence of two methyl groups in the bridge-head positions of compound **26** is crucial in these annulation reactions. These groups play a vital role in preventing undesired side reaction pathways, as their absence would lead to the failure in the formation of the desired target products. Upon comparing the UV–vis absorbance graphs of compounds **28** and **29**, POA **29** (λ_max_ = 500 nm), which was obtained through the aromatization of compound **28**, exhibited a significant bathochromic shift. These observations further support the hypothesis that electrons can indeed be delocalized through the 4-membered ring system in the POAs.

**Scheme 7:**
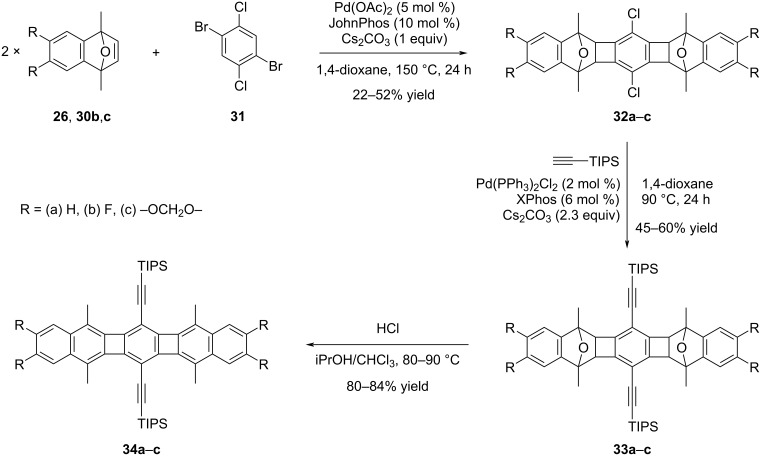
Synthesis of bisphenylene-containing structures **34a**–**c**.

Following their work reported in 2017 [38], Xia and his group directed their attention towards refining the electronic properties of POAs through structural variations [39]. Using catalytic annulation reactions involving 1,4-dibromo-2,5-dichlorobenzene (**31**) and substituted oxanorbornenes (**26** and **30b**,**c**), products **32a**–**c**, with R groups representing (a) H, (b) F, and (c) –OCH_2_O–, were successfully synthesized, albeit with moderate yields (Scheme 7).

Following that, compounds **32a**–**c** underwent derivatization through Sonogashira cross-coupling reactions with alkynes featuring different protecting groups such as TIPS, TES, and TIBS. Scheme 7 illustrates the derivatization process using one of the chosen examples, specifically the TIPS group. Accordingly, the cross-coupling products **33a**–**c** were obtained in yields ranging between 45% and 60%. The last step of the sequential reactions is the aromatization step and the target POAs **34a**–**c** were obtained in yields between 80–84%. UV–vis investigations conducted on compounds **34a**–**c** revealed absorption bands that align well with acene structures. While **34a** and **34b** displayed nearly identical absorption profiles with a maximum absorption at λ_max_ = 515 nm, the incorporation of donor groups in compound **34c** led to a noteworthy bathochromic shift with a maximum absorption at λ_max_ = 534 nm. Additionally, it was reported that all three compounds, **34a**–**c**, demonstrated remarkable stability, showing no signs of degradation over an extended period when kept in the dark, both in solid form and in solution under air. In the final phase of the study, the authors investigated the charge-transport properties of compond **34a** in OFET. Since the charge-transport properties are significantly affected by the molecular packing, they have modified compound **34a** using different protecting groups. In this context, triethylsilyl (TES), triisopropylsilyl (TIPS), and triisobutylsilyl (TIBS) groups were incorporated into the structure considering the increased dimensions. Thus, derivatives **34a**-TES and **34a**-TIPS showed hole mobilities of 0.075 cm^2^ V^−1^ s^−1^ and 0.19 cm^2^ V^−1^ s^−1^, respectively, while the highest value was noted with **34a**-TIBS at 0.52 cm^2^ V^−1^ s^−1^.

**Scheme 8:**
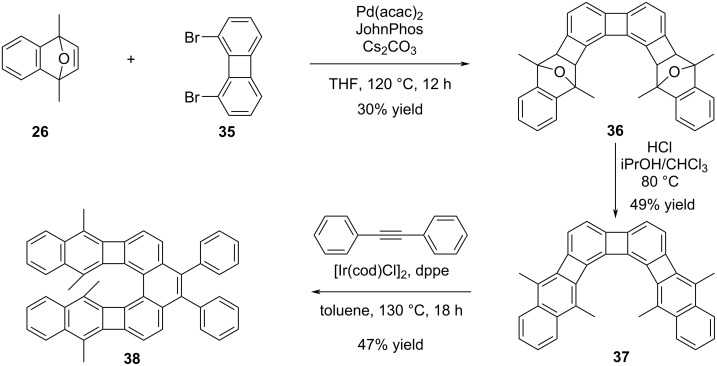
Synthesis of curved PAH **38** via Pd-catalyzed annulation and Ir-catalyzed cycloaddition reactions.

Later on, Xia and his colleagues demonstrated that curved PAH structures can be synthesized by employing their own developed palladium-catalyzed arene–oxanorbornadiene annulation reactions [40]. This study involved converting PAHs obtained through annulation and aromatization steps into curved PAH structures using metal-catalyzed cycloaddition reactions pioneered by Vollhart [41] and Kotora [42]. Unlike previous studies that reported cycloadditions from bay regions of [N]phenylenes, metal-catalyzed cycloadditions with diphenylacetylene occurred exclusively in the non-bay region, which allowed for straightforward syntheses of curved structures. Moreover, the presence of methyl groups in the structure facilitated the controlled activation of desired cyclobutadiene units, enabling precise modifications. A selected example from the study is summarized in Scheme 8. The angular structure **36** was prepared through annulation reaction between oxanorbornene **26** and 1,8-dibromobiphenylene (**35**), followed by aromatization via treatment with HCl in CHCl_3_ and iPrOH, resulting in the formation of compound **37** in 49% yield. In the final step, Ir-catalyzed cycloaddition reaction with diphenylacetylene (tolane) led to PAH **38** in 47% yield.

According to the X-ray analysis results, it is evident that the structure of compound **38** is far from planarity, and the phenanthrene moiety exhibits a dihedral angle of approximately 22°. Upon comparing the UV–vis spectra of the angular structures **37** and **38**, it was observed that after the Ir-catalyzed cycloaddition reaction, the λ_max_ of product **38** considerably blue shifted in comparison to the λ_max_ of **37**.

**Scheme 9:**
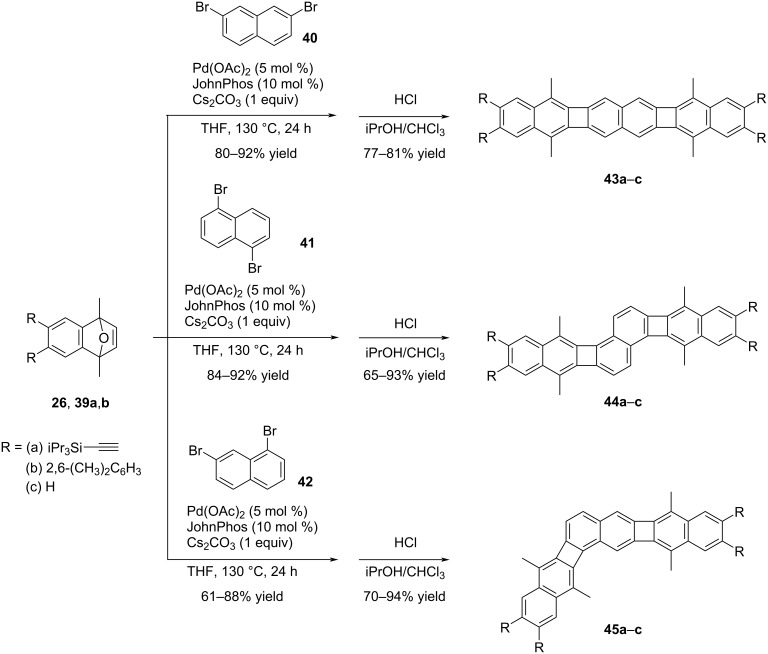
Synthesis of [3]naphthylenes.

Xia et al. also conducted a synthesis of [3]naphthylene regioisomers through Pd-catalyzed annulation reactions, employing 2,7-, 1,5-, and 1,7-dibromonaphthalenes (Scheme 9) [43].

These annulation reactions involving 2,7-, 1,5-, and 1,7-dibromonaphthalene with different benzoxanorbornadienes (R = (a) TIPSA, (b) 2,6-(CH_3_)_2_C_6_H_3_, (c) H), followed by aromatization in acidic conditions, resulted in the formation of three [3]naphthalene regioisomers **43a**–**c**, **44a**–**c**, and **45a**–**c** with excellent yields of up to 94%. The synthesized PAHs **43a**, **44a**, and **45a** with diverse geometries exhibited interesting absorption and emission characteristics, making them highly intriguing for further study and potential applications. Among the regioisomers in the series, the linear isomer **43a** displayed the highest quantum yield (Φ_em_ = 0.64). Additionally, its absorption and emission max values (λ_max_ and λ_max,em_) were determined to be 476 and 477 nm, respectively. Compound **44a** exhibited no alteration in the absorption maxima; however, there was a considerable bathochromic shift in the emission maxima (λ_max_ = 476 nm and λ_max_,_em_ = 587 nm for **44a**). In contrast to compound **43a**, Φ_em_ of compound **44a** decreased significantly to 0.08. Likewise, compound **45a** (λ_max_ = 402 nm*,* λ_max,em_ = 524 nm, Φ_em_ = 0.16) also demonstrated a substantially reduced quantum yield when compared to compound **43a**. Theses regioisomers with varying geometric structures provided strong evidence that the antiaromaticity of the cyclobutadiene (CBD) ring can be tuned up with appropriate structural designs. NICS (nucleus independent chemical shift) analysis revealed that the linear structures within the series exhibited reduced antiaromaticity in the CBD ring compared to other structural arrangements.

**Scheme 10:**
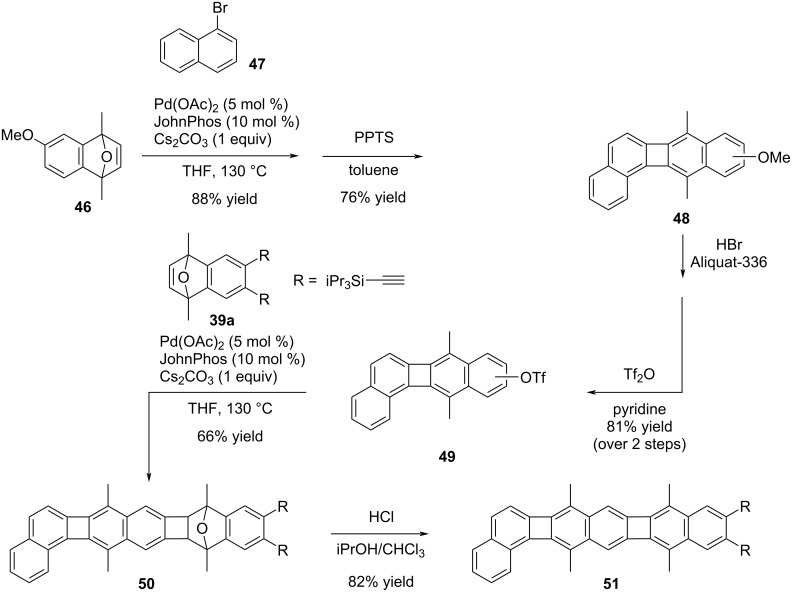
Sequential Pd-catalyzed annulation reactions.

By employing a methoxy group on the oxonorbornene, the methyl ether moiety can be removed and the phenol subsequently converted to a triflate. The Pd-catalyzed annulation approach can be conducted sequentially, facilitating the synthesis of polyaromatic hydrocarbons, particularly unsymmetrical ones (Scheme 10) [44]. In this way, compound **48** was synthesized through a two-step process involving the Pd-catalyzed annulation between compounds **46** and **47**, followed by aromatization in the presence of PPTS. The methoxy group was then cleaved using HBr in Aliquat-336, and the resulting product was converted to the triflate under basic conditions, resulting in a regioisomeric mixture of **49** with a yield of 81% over the two steps. To obtain the PAH **51**, a second Pd-catalyzed annulation and subsequent aromatization of **50** were performed in an acidic medium. Ultimately, the desired compound, PAH **51** bearing TIPS groups, was obtained in 82% yield as the final product. UV–vis and fluorescence studies were conducted to gather insights into the optoelectronic characteristics of PAH **51**. The obtained data revealed that the synthesized compound possesses a unique absorption and emission profile, highlighting its distinctive optical properties (λ_max_ = 470 nm, λ_max,em_ = 470 nm, Φ_em_ = 0.12).

### Biphenylene-containing azaacenes

**Scheme 11:**
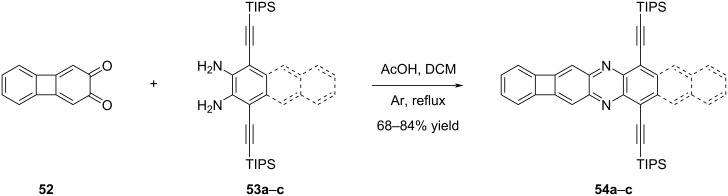
Synthesis of biphenylene-containing unsymmetrical azaacenes **54a**–**c**.

Bunz and co-workers incorporated biphenylene units into azaacene structures to enhance their stability [45]. Initially, their early attempts focused on synthesizing unsymmetrical azaacenes containing biphenylene units. Through the condensation of *ortho*-diamine compounds **53a**–**c** derived from benzene (**53a**), naphthalene (**53b**), and anthracene (**53c**) with compound **52**, the desired target azaacenes **54a**–**c** were successfully obtained in yields ranging from 68% to 84% (Scheme 11). As anticipated, there was a notable red shift observed from **54a** to **54c**, which can be attributed to the expansion of the acene structure (λ_max_ = 441 nm, λ_max,em_ = 444 nm for **54a**; λ_max_ = 541 nm, λ_max,em_ = 550 nm for **54b**; λ_max_ = 648 nm, λ_max,em_ = 655 nm for **54c**).

**Scheme 12:**
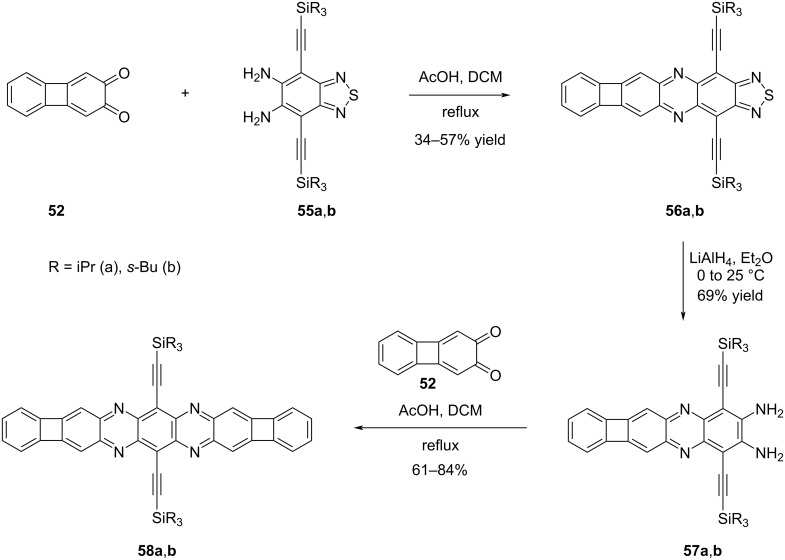
Synthesis of biphenylene containing symmetrical azaacenes **58a**,**b**.

Next, the Bunz group focussed on symmetric azaacenes, which present a greater challenge in synthesis compared to their non-symmetric counterparts (Scheme 12) [46]. This complexity arises from factors such as limited substrate versatility and the difficulty in incorporating solubilizing groups into the symmetric azaacene framework. In the initial stage, the condensation reactions between biphenylene-2,3-dione (**52**) and diaminothiadiazoles **55a**,**b** resulted in the formation of polycyclic structures **56a** and **56b** containing thiadiazole units, in yields of 57% and 34%, respectively. Subsequently, by selectively cleaving the thiadiazole ring using LiAlH_4_, followed by further condensation reaction with dione **52**, the desired symmetric azaacenes **58a** and **58b** with R groups representing (a) isopropyl and (b) *sec*-butyl, were successfully obtained in 84% and 61% yields, respectively. The incorporation of a biphenylene group into the azaacene structure did not result in any significant impact on the electrochemical properties. The electrochemical analysis revealed the presence of two reduction potentials commonly observed in azaacenes, suggesting that the modification did not alter this characteristic feature. For compound **58a**, λ_max_ was observed at 600 nm, and λ_max,em_ was at 614 nm. On the other hand, for compound **58b**, λ_max_ was found at 606 nm, and λ_max,em_ occurred at 616 nm. Based on the photophysical properties of compounds **54a** and **54b**, which were synthesized in the previous study (Scheme 11), it is evident that the addition of the second biphenylene-fused pyrazine group to the structure leads to a substantial red shift towards the NIR region. This observation indicates that the incorporation of biphenylene-containing groups has a positive impact on the optoelectronic properties of these structures, while also promoting their stability.

**Scheme 13:**
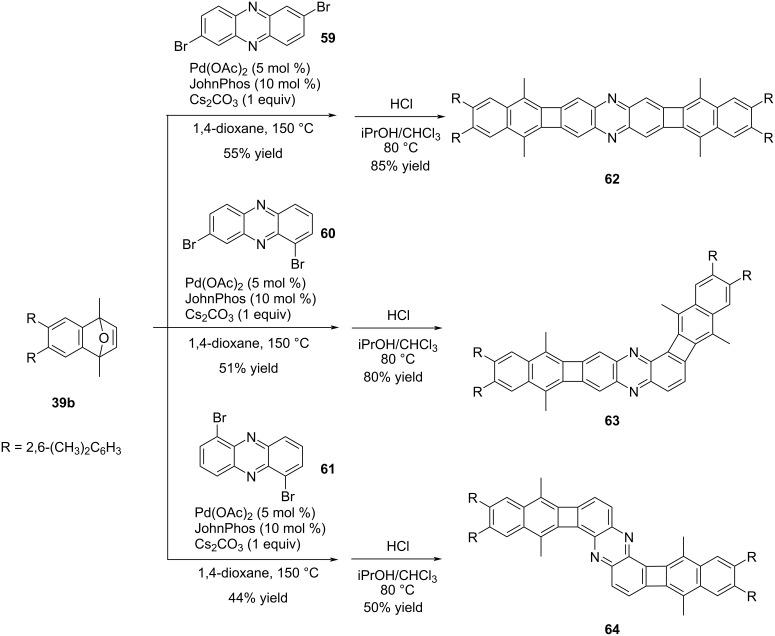
Synthesis of azaacene analogues **62**–**64**.

With appropriate substrate choices, Xia and co-workers were able to apply the Pd-catalyzed annulation strategy that they developed for the synthesis of biphenylene-containing azaacene structures [47] (Scheme 13). The methodology used in this study closely resembled their previous work (Scheme 9) [43].

The method used offered a distinct advantage compared to previous approaches [45], as it enabled the synthesis of not only linear azaacene structures but also non-linear structures as shown in Scheme 13. This versatility allowed for a broader range of conjugated molecules to be successfully prepared. The target azaacene analogues **62**–**64**, featuring 2,6-(CH_3_)_2_C_6_H_3_ groups were obtained in yields ranging from 50 to 85% through a series of steps. First, Pd-catalyzed annulation reactions of oxanorbornene **39b** and dibromophenazine derivatives **59**–**61** were carried out. Subsequently, aromatization reactions were performed in the presence of HCl to yield the desired products. The conjugated structures with distinct geometries demonstrated significant absorption and fluorescence characteristics. Among the synthesized compounds, linear derivative **62** exhibited the most intense absorption peak (λ_max_ = 503 nm), whereas the other two compounds **63** and **64** possessed slightly blue-shifted absorption maxima at 487 nm and 501 nm, respectively. When the fluorescence properties of compounds **62**, **63**, and **64** were examined, it was observed that compound **62** exhibited strong fluorescence, making it an excellent fluorophore (λ_max,em_ = 508 nm, Φ_em_ = 0.58) . On the other hand, compound **63** showed weaker fluorescence compared to **62** (λ_max,em_ = 666 nm, Φ_em_ = 0.07), and compound **64** displayed almost no fluorescence emission (λ_max,em_ = 497 nm, Φ_em_ = NA).

### Naphthazarin–biphenylene hybrid structures

**Scheme 14:**
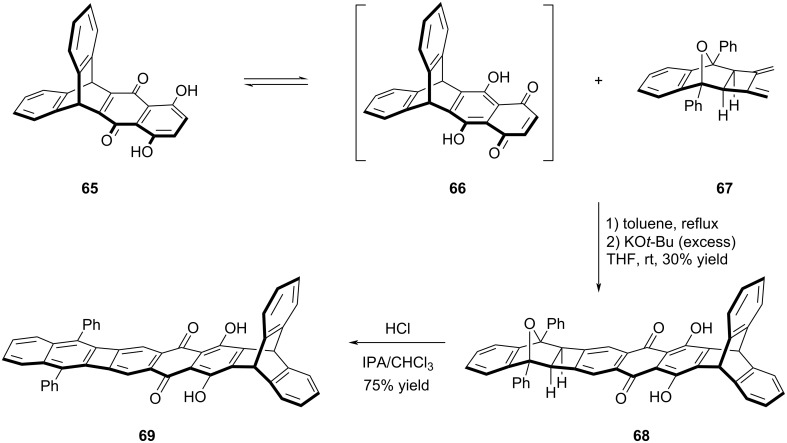
Synthesis of POA-type structure **69**.

Taking the advantage of naphthazarin's bifunctional Diels–Alder reactivity, Swager and his team succeeded in the synthesis of POA-type structures incorporating naphthazarin and triptycene units (Scheme 14) [48]. Naphthazarin derivatives are known to complex with boron moieties and metals to form electron-poor acene units. Through the Diels–Alder reaction involving dienophile **66**, which was formed via the tautomerization of compound **65**, and diene **67**, compound **68** was successfully obtained in 30% yield. In the final step, the target POA-type structure containing naphthazarin **69** was synthesized by aromatization under acidic conditions. The attempts to conduct complexation experiments using BF_3_·OEt_2_ to obtain a stable BF_2_ complex were unsuccessful, primarily due to solubility problems encountered with substrate **69** and its BF_2_ complex. Based on the UV–vis analysis of compounds **65** and **69**, it was observed that compound **65** displayed a low-energy absorption band at λ_max_ = 568 nm, whereas compound **69** exhibited absorption at λ_max_ = 557 nm. Unfortunately, the unsuccessful BF_2_ complexation step to isolate stable BF_2_ complex of **69**, likely attributable to solubility issues encountered with substrate **69**, prevented the incorporation of the acene backbone into the structure. Consequently, the anticipated red-shift in the absorption spectrum could not be observed as expected.

### Boron-doped phenylene-containing oligoacenes

**Scheme 15:**
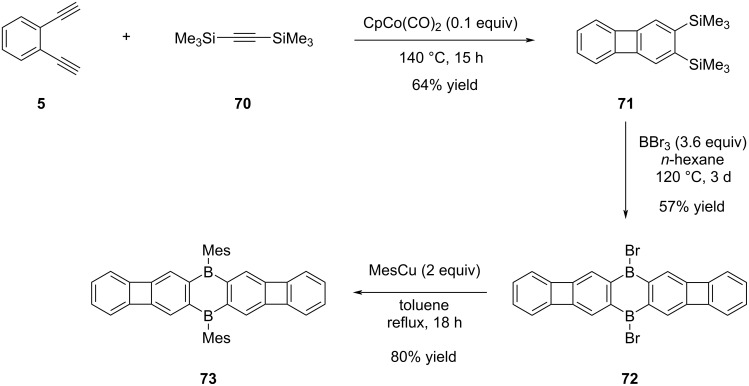
Synthesis of boron-doped POA **73**.

Following the evaluation of the POA concept combined with azaacenes and naphthazarin derivatives, the impact of boron doping on the optoelectronic properties of POA structures was also targeted [49]. In the study, the envisaged POA **73** was successfully synthesized in three steps (Scheme 15). The initial step involved the synthesis of compound **71** in 64% yield using a cobalt-catalyzed cyclotrimerization reaction between 1,2-diethynylbenzene (**5**) and bis(trimethylsilyl)acetylene (**70**), a method commonly employed in [N]phenylene synthesis. Subsequently, treatment of compound **71** with excess BBr_3_ in *n*-hexane, dibrominated intermediate **72** was obtained in 57% yield. In the final step, mesitylation was conducted utilizing mesitylcopper, leading to the successful access of the desired boron-doped POA **73** in 80% yield.

The red-colored POA **73** is noted for its absence of well-resolved absorption bands above 450 nm. Instead, the compound's color is attributed to plateau-shaped weak absorption bands, extending to approximately 570 nm. Surprisingly, POA **73** exhibited nonfluorescent behavior, in contrast to previously known POAs described in the literature. The hypothesis suggesting that this phenomenon is a result of non-radiative deactivation was corroborated by detailed computational chemistry studies.

**Scheme 16:**
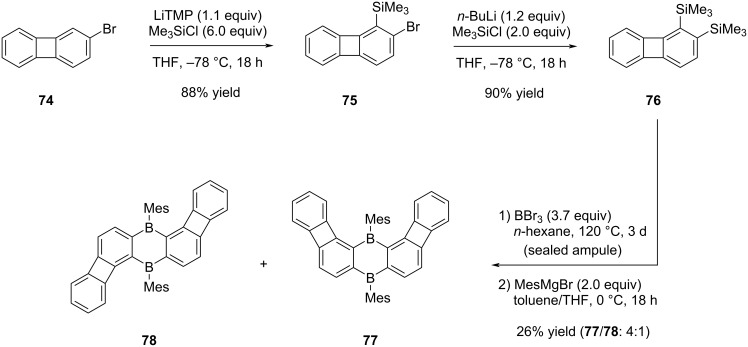
Synthesis of “v”- and “z”-shaped B-POAs **77** and **78**.

**Scheme 17:**
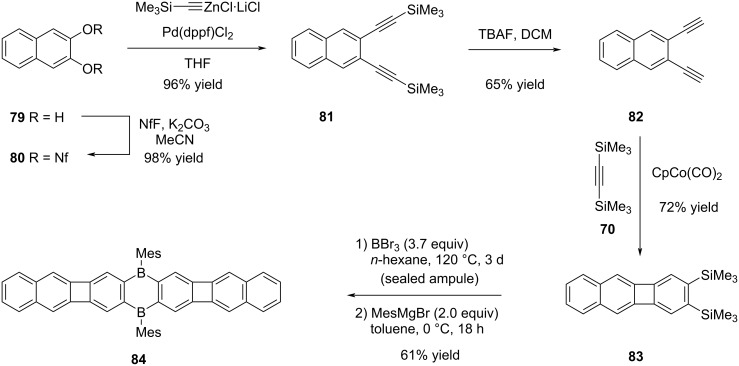
Synthesis of boron-doped extended POA **84**.

In continuation of the aforementioned work by Wagner and co-workers [49], they have synthesized also "v" and "z"-shaped POAs, wherein biphenylene groups are angularly incorporated into the 1,4-dibora-2,5-cyclohexadiene structure (Scheme 16) in addition to a π-extended linear POA (Scheme 17) [50]. The preparation of v- and z-shaped POAs **77** and **78** was carried out starting from 2-bromobiphenylene (**74**). Initial steps involved *ortho*-directed lithiation and subsequent treatment with Me_3_SiCl, resulting in the formation of compound **75**. Further transformation through lithium–halogen exchange, followed by reaction with Me_3_SiCl, yielded bis(trimethylsilylated) intermediate **76** in a 90% yield. By utilizing the conditions outlined in the prior investigation [49], compound **76** was subjected to a reaction with BBr_3_. In the final step, the integration of mesityl groups into the molecular structure, resulted in the production of both compounds **77** and **78**, adopting v and z-configurations, respectively, in a 4 to 1 ratio.

The synthesis of linear compound **84**, characterized by a more extensive conjugated system in comparison to the boron-modified POA **73** outlined in Scheme 15, was accomplished through a sequence of five steps, starting from 2,3-dihydroxynaphthalene (**79**, Scheme 17). Following the successful synthesis of the nonaflate during the initial phase starting from **79**, the subsequent step involved the synthesis of compound **81** through the utilization of the Negishi cross-coupling reaction and then the removal of TMS groups from this intermediate was achieved using TBAF, resulting in the formation of diyne **82** in 65% yield. The progression towards the synthesis of biphenylene-containing substrate **83** was achieved through a Co-mediated alkyne trimerization process. Finally, the synthesis of the targeted boron-doped extended POA **84** was carried out with a yield of 61%, following a series of reactions including cyclocondensation with BBr_3_ and mesitylation.

Since the "v" and "z"-shaped POAs could not be separated by physical methods, it was not possible to study their photophysical properties separately. Nonetheless, comparison of the properties of POAs **73** and **84** has provided essential data. Closely resembling compound **73**, the red color of POA **84** is attributed to an extensively broad absorption band, with an onset wavelength at around 570 nm. Although POA **73** does not exhibit any fluorescence characteristics, compound **84** displays a red emission, which can be attributed to the presence of two benzene rings integrated within its structure (λ_max,em_ = 646 nm, Φ_em_ = 0.12). The fluorescence properties of the mixture **77**/**78** were markedly enhanced in comparison to those of linear POAs **73** and **84**, owing to the distinctive geometric arrangement inherent in their structures (λ_max,em_ = 506 nm, Φ_em_ = 0.65).

### On-surface synthesis of phenylene-containing oligoacenes

The POA syntheses discussed in this review thus far have predominantly involved solution chemistry. However, recent reports have demonstrated the feasibility of producing biphenylene-containing polycyclic aromatic compounds through on-surface chemistry techniques. One of the initial examples was documented by Fasel and Meunier in 2017 [51]. In their study, they effectively synthesized POA **87** utilizing 2,3-dibromotetracene (**85**) as substrate at a temperature of 430 K, employing ultra high vacuum conditions on Ag(111).

**Scheme 18:**
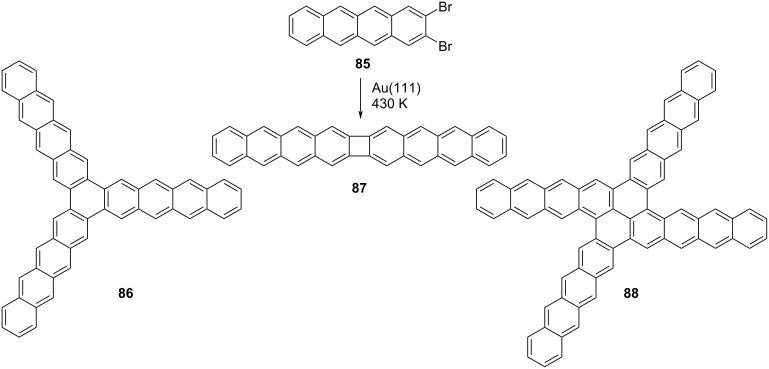
Ag(111) surface-catalyzed synthesis of POA **87**.

Subsequent scanning tunneling microscopy (STM) analyses unveiled not only the linear POA **87** resulting from surface-catalyzed formal [2 + 2] cycloaddition reactions but also the emergence of tetracene trimer **86** and tetramer **88** stemming from [2 + 2 + 2] cycloaddition reactions (Scheme 18). It is proposed that an aryne intermediate is formed after thermal activation and that the observed end products are formed from arynes via [2 + 2] and [2 + 2 + 2] cycloadditions.

**Scheme 19:**
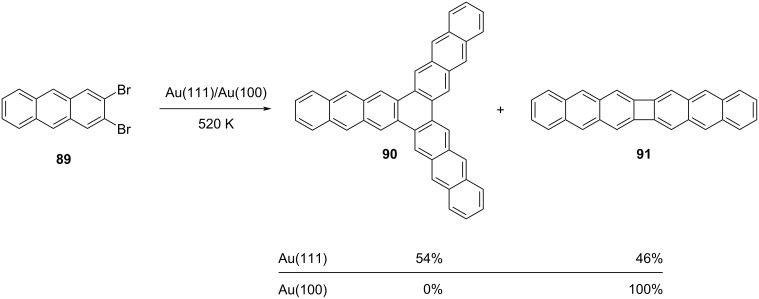
Au(100) and Au(111) surface-catalyzed synthesis of POA **91**.

In a closely related study conducted by Grill et al., the behavior of 2,3-dibromoanthracene (**89**) was examined on two distinct surfaces [Au(100) and Au(111)] (Scheme 19) [52]. Notably, on the Au(111) substrate, nearly equivalent quantities of dimer **91** resulting from a [2 + 2] cycloaddition and trimer **90** formed via a [2 + 2 + 2] cycloaddition pathway were observed. In contrast, when applied to Au(100), only the dimer structure **91** was generated through the [2 + 2] cycloaddition process.

**Scheme 20:**
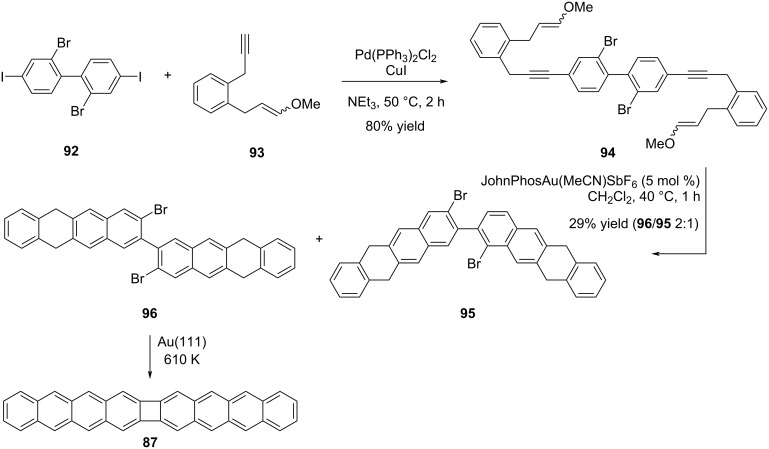
Au(111) on-surface synthesis of POA **87**.

In a recent study in this field, Izydorczyk et al. were able to selectively synthesize compound **87** through a hybrid approach involving the integration of both solution and surface chemistry techniques [53]. The key compound **96** to be used in the synthesis of POA **87** was synthesized in two steps. In the first step, **94** was obtained using a double Sonogashira cross-coupling reaction, followed by a Au(I)-catalyzed [4 + 2] cycloaddition reaction to afford the target substrate **96** and its regioisomer **95** in a 2:1 ratio (Scheme 20). POA **87** was obtained on Au(111) at 610 K after Ullmann-type coupling and aromatic dehydrogenation of compound **96**. Apart from these studies, the synthesis of smaller POA units has been accomplished by similar methods starting from naphthalene percursors on the Ag(111) surface [54]. The synthesis of nanoribbons containing biphenylene units was also achieved on the Ag(111) surface at 475 K by using 2,3,8,9-tetrabromotetracene as the substrate [55].

## Conclusion

In conclusion, even though considerable time has elapsed since its initial synthesis in 1941, biphenylene continues to be a significant synthetic target, with a notable increase in research activity in recent times. Despite this long history, the synthetic methods used in the synthesis of biphenylene are largely limited to flash vacuum pyrolysis, [2 + 2] cycloaddition, [2 + 2 + 2] cycloaddition, and the Ullmann reaction. This shows how open this field is to further development and indicates the significant potential for new methodologies to be developed. In particular, the utilization of biphenylene units for stabilizing polycyclic aromatic compounds, along with the instances elucidated in this review where the electron delocalization occurs through the 4-membered ring, amplifies the intrinsic value of these structures. Methods that combine heteroatoms with biphenylene moieties have also been employed to alter the electronic characteristics and enhance the stability of polycyclic aromatic (POA) structures. Over the recent years, it has been shown that synthetically demanding biphenylene-containing polycyclic aromatic compounds can be achieved in a controlled manner not only by solution chemistry but also by on-surface chemistry. All these results, which are open for further development, confirm that biphenylene structures are very important synthetic units and will be used as tools for the synthesis of more complex structures in the future.
